# Digital subtraction radiographic analysis of the combination of bioabsorbable membrane and bovine morphogenetic protein pool in human periodontal infrabony defects

**DOI:** 10.1590/S1678-77572010000400010

**Published:** 2010

**Authors:** Maria do Carmo Machado GUIMARÃES, Euloir PASSANEZI, Adriana Campos Passanezi SANT’ANA, Sebastião Luiz Aguiar GREGHI, Mario TABA JUNIOR

**Affiliations:** 1 DDS, MSc, PhD, Adjunct Professor of Periodontics, Periodontics Division, University of Brasilia, Brasília, DF, Brazil.; 2 DDS, MSc, PhD, Full Professor, Department of Prosthodontics, Bauru School of Dentistry, University of São Paulo, Bauru, SP, Brazil.; 3 DDS, MSc, PhD, Assistant Professor, Department of Prosthodontics, Bauru School of Dentistry, University of São Paulo, Bauru, SP, Brazil.; 4 DDS, MSc, PhD, Assistant Professor, Department of Oral and Maxillofacial Surgery and Traumatology and Periodontology, Ribeirão Preto Dental School, University of São Paulo, Ribeirão Preto, SP, Brazil.

**Keywords:** Alveolar bone loss, Regeneration, Radiography

## Abstract

**Objectives:**

This study assessed the bone density gain and its relationship with the periodontal clinical parameters in a case series of a regenerative therapy procedure.

**Material and Methods:**

Using a split-mouth study design, 10 pairs of infrabony defects from 15 patients were treated with a pool of bovine bone morphogenetic proteins associated with collagen membrane (test sites) or collagen membrane only (control sites). The periodontal healing was clinically and radiographically monitored for six months. Standardized presurgical and 6-month postoperative radiographs were digitized for digital subtraction analysis, which showed relative bone density gain in both groups of 0.034 ± 0.423 and 0.105 ± 0.423 in the test and control group, respectively (p>0.05).

**Results:**

As regards the area size of bone density change, the influence of the therapy was detected in 2.5 mm^2^ in the test group and 2 mm^2^ in the control group (p>0.05). Additionally, no correlation was observed between the favorable clinical results and the bone density gain measured by digital subtraction radiography (p>0.05).

**Conclusions:**

The findings of this study suggest that the clinical benefit of the regenerative therapy observed did not come with significant bone density gains. Long-term evaluation may lead to a different conclusions.

## INTRODUCTION

The improvement in the clinical parameters shown by the reduction in probing depth, clinical attachment gain and absence of bleeding on probing must not necessarily be interpreted as regeneration. The nature of neoformed tissue and the occurrence of regeneration can only be defined by means of microscopic analysis^25^.

Conventional radiographic analysis presents limitations as regards image quality, and is more subject to error, especially if the images were not standardized. Similarly, it only allows alterations to be visualized when the loss attains 30% to 60% of mineral bone content. On the other hand, the radiographic subtraction technique allows changes in density to be quantified by comparing alterations in the image with reference structures, and by detecting bone alterations of 5%, with over 90% of sensitivity and specificity^9^.

The properties of osteoconduction and integration into bone tissue^26^ have enabled the use of xenogenous grafts derived from bovine bone (BDX) in regenerative periodontal therapy^11,20,24,30^. The material is slowly reabsorbed by osteoclastic activity^2^. Results from histological and clinical studies have demonstrated that BDX is very well tolerated, and up to now, no allergic reactions related to the material have been reported^4,21,24^. Furthermore, since its supply is unlimited, no donor site is required.

The use of bovine bone matrix in regenerative therapy is based on the homology proved by DNA cloning of human and bovine bone morphogenetic proteins (BMPs)^23^. As the BMPs concentrate the osteogenic potential generically attributed to the matrix, in the present study a pool containing the extract of these proteins with the largest quantities of different types of these proteins was applied.

The aim of this study was to evaluate the bone density gain by means of radiographic subtraction, in infra-osseous defects treated with a pool of bovine BMPs associated with reabsorbable bovine collagen membrane. Furthermore, the aim was to establish the correlation between the change in density of the treated defects and the variation in the probing depth and attachment level measurements. The clinical results as regards the reduction of probing depth and improvement in attachment level have previously been detailed^16^.

## MATERIAL AND METHODS

### Study design and clinical procedures

Fifteen patients of both genders (age range: 26 to 57 years; mean age: 36.06 years) were selected at the Periodontics Clinic of Bauru School of Dentistry, University of São Paulo, Brazil. The possible types of treatment, associated risks and benefits were explained to the patients and all signed the informed consent form agreeing to the treatment. The study was approved by the local Research ethics Committee.

The inclusion criteria were as follows: 1) good systemic health; 2) no use of drugs such as antibiotics, corticoids, chemotherapeutic agents, or immunological modulators that might alter the response of the oral tissue; 3) no smoking; 4) radiographic evidence of at least one pair of interproximal infrabony defects with 2 or 3 walls located in the same jaw (maxilla or mandible), and the same type of tooth (premolars or molars), without involvement of the furcations; attachment level ≥ 5 mm; tooth mobility < class II.

After selection, all patients underwent initial therapy, consisting of oral hygiene instruction and scaling and root planing. After extra and intraoral asepsis of the surgical field, full-thickness mucoperiosteal flaps were raised by means of buccal and lingual intracrevicular incisions up to at least two adjacent teeth - one mesial and one distal, followed by removal of the granulation tissue and bone debridement. Defects were divided into test and control sites in a split-mouth design. The test sites were treated with a pool of bone morphogenetic proteins (Genpro, Baumer S.A., Bauru, SP, Brazil ) obtained from fetal bovine bone matrix with resorbable hydroxyapatite carrier (HA) (BMP HA in a 1:20 ratio), with the addition of demineralized lyophilized bovine bone matrix (BM) (Mo-Gen-Ox, Baumer S.A., Bauru, SP, Brazil) in a 1:1 ratio in sterile saline solution, which was applied in the test sites.

The control sites were treated with BM and HA (MB-HA), in a 1:1 ratio, therefore excluding only the pool of bovine BMPs. After filling the defects with the respective biomaterials, both the test and control defects were covered with a resorbable membrane obtained from bovine cortical bone (Genderme, Baumer S.A.). For all patients, 100 mg of oral doxycycline once a day during 14 days were prescribed and they were instructed to rinse twice daily with 0.12% digluconate chlorhexidine during 6 weeks. All patients were seen for professional prophylaxis weekly during the first 6 weeks and then monthly until 6 months postoperatively. At 6 months after surgery, new clinical measurements were taken and new standardized radiographs were obtained.

### Radiographic Procedures

**Figure 1:**
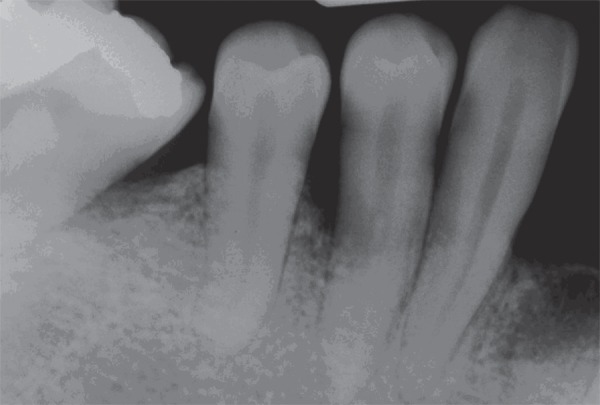
Preoperative radiograph

**Figure 2:**
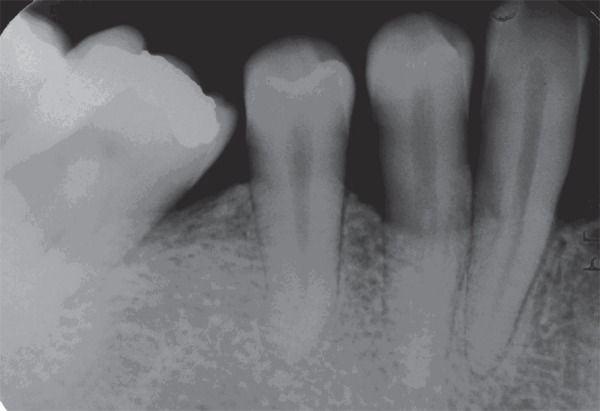
Six-month postoperative radiograph

**Figure 3:**
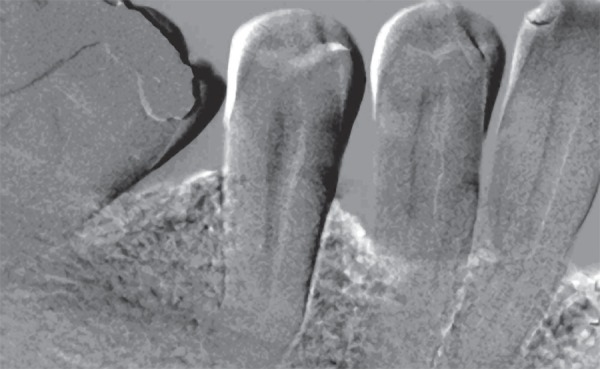
Subtraction radiograph showing a bone density gain on the mesial aspect of the mandibular first molar and mandibular first premolar

Periapical radiographs were taken with the aid of an acrylic device (Resapol T 208, Outline Fiberglass, São Vicente, SP, Brazil) made by duplication of a positioner-matrix (Dentsply Rinn, elgin, IL, USA). The device was coupled to a bite block made of acrylic material (Duralay, Reliance Dental Mfg. Co, Worth, IL, USA) at each site to retain reproducible projection with exposure settings of 70 KV at 8 mA for 1.0 second. Radiographs were taken at baseline and 6 months (Figures 1 and 2). The radiographic films were developed in an automatic radiograph processing machine (Perio-pro II, Air Technique Inc., Melville, NY, USA). The radiographic pairs were scanned and digitized using a 35-mm slide scanner (Polaroid SprintScan, Polaroid Corporation, Simi Lalley, CA, US) connected to a Pentium 100 Mhz PC (Intel Corporation, Santa Clara, CA, USA). The baseline presurgical and postoperative images were aligned by the selection of common reference points^10^. The images were subtracted and analyzed using diagnostic subtraction radiography software, IDL- Interactive Data Language v.5.0 (Research Systems, Boulder, CO, US) developed for this purpose (Figure 3). The routines of the program work on the platform of the envi 3,5 (ITT Visual Information Solutions, Boulder, CO, USA), software solution for processing and analyzing images that allows development.

Following selection of an area of interest on the images, the post-operative film was subtracted from the presurgical film, with the software program compensating for any geometric projection and film contrast differences between the pairs of images. Changes between films were depicted as a darkened area for loss of alveolar bone mass, a neutral gray for no change in alveolar bone mass, and a lightened area for an increase in alveolar bone mass^10^. All subtracted images were subsequently uploaded into the Image Tool software (The University of Texas, Health Science Center in San Antonio, San Antonio, TX, USA) to calculate the average density of areas that showed changes in subtraction and to quantify these areas. In order to determine the changes in density in the subtracted image, this same image was set up as a reference and the value of the density of a neutral area, ie, an area that had supposedly not changed during the study, like the dental enamel, for example, was used as a "standard area". After obtaining this value, the rule of three was applied to check the value of change in the area of the defect. The quantification of the changed area provided by the Image Tool software was converted into millimeters.

### Data analysis

The t test (p<0.05) was applied to indicate whether the differences with regard to the area (quantification in mm) of alteration in bone density of the test and control sites were statistically significant as well as to verify the differences between the means of alteration in density of the test and control sites. Pearson’s correlation (p<0.05) was applied to verify whether there was correlation between the alteration in bone density and variation of probing depth and attachment level measured at baseline and after 6 months.

## RESULTS

According to Table 1, the t-test indicated that the differences between the test and control groups with regard to quantification of the area of change in density were not statistically significant. The t-test indicated that there was no statistically significant difference between the test and control sites with regard to alteration in density by the radiographic subtraction analysis.

**Tabela 1:** Quantification of the areas and mean change in bone density of the test and control sites by the t-test (p<0.05)

	**Test**	**Control**	**Difference**	**t**	**p value**
	**Mean (SD)**	**Mean (SD)**			
					
Quantification of the areas	576.89 (309.106)	507.77 (352.85)	69.128	0.877	0.395 ns
	(≅25.08 mm^2^)	(≅22.07 mm^2^)			
Mean change in bone density	0.034 (0.423)	0.105 (0.423)	-0.071	0.839	0.415 ns

ns- Non-significant differences (P>0.05). SD= Standard Deviation

The variation in probing depth and attachment level at the buccal and lingual faces, between the periods measured at baseline and 6 months, presented in Table 2, were correlated with the change in density (Table 3), and it can be observed that the alteration in density presented no correlation with the variation in probing depth and attachment level at the buccal and lingual faces, in both test and control sites.

**Tabela 2:** Variation in probing depth (PD) and attachment level (AL) at the buccal (B) and lingual (L) faces of the test and control sites, between the 6 months (6m) and baseline (bas) periods by the t-test

**Variation**	**Face**	**Test**		**Control**		**p value**
		**mean**	**sd**	**mean**	**sd**	
						
PD 6m-bas	B	-1.633	1.141	-1.933	1.347	0.443 ns
	L	-1.933	0.961	-2.000	1.511	0.886 ns
AL 6m- bas	B	-1.600	1.168	-1.033	1.245	0.165 ns
	L	-1.466	0.972	-1.300	1.114	0.591 ns

ns- Non-significant differences (P>0.05)

**Tabela 3:** Correlation of the change in density and variation in probing depth and attachment level at the buccal (B) and lingual (L) faces of the test and control sites by the Pearson's correlation test (p<0.05), between the 6 months (6m) and baseline (bas) periods

**Group**	**Correlation**	**r**	**p value**
			
	Density x PD (B) 6m-bas	-0,2	0.473 ns
Test	Density x PD (L) 6m-bas	-0,15	0.602 ns
	Density x AL (B) 6 m-bas	-0,2	0.471 ns
	Density x AL (L) 6m-bas	-0,25	0.368 ns
			
	Density x PD (B) 6m-bas	0,11	0.691 ns
Control	Density x PD (L) 6m-bas	-0,34	0.212 ns
	Density x AL (B) 6 m-bas	0,7	0.804 ns
	Density x AL (L) 6m-bas	-0,42	0.117 ns

ns- Non-significant differences (P>0.05)

## DISCUSSION

The extracellular matrix of mineralized tissues constitutes a rich reservoir of morphogenetic proteins and other growth factors. The osteogenic properties of the BMPs in this matrix have led to their evaluation as possible adjuncts in regenerative periodontal therapy. The findings of Urist^27^ (1965) and later of Reddi and Huggins^22^ (1972) demonstrated that devitalized and demineralized bone matrix is capable of inducing heterotopic osteogenesis. Special preparatory procedures, such as eliminating cells by autolysis and extraction of matricial antigens have enabled this matrix to be used in the induction of new bone formation^28,29^. The osteogenic and osteoinductive properties of BMPs present within the organic matrix induce the formation of new bone tissue.

In several reports, it was found that infra-osseous defects were filled with neoformed bone tissue during re-entry surgery performed at 6 months postoperatively^1,4,18^. In some studies that treated infra-osseous defects with absorbable barriers^8,18^ or other regenerative therapies^20^, surgical re-entry was complemented by the radiographic subtraction technique to evaluate bone density gain.

With the object of defining the nature of neoformed tissue in infra-osseous defects, Sculean, et al.^25^ (1999) performed a microscopic analysis of teeth indicated for extraction, which had been submitted to regenerative therapy. Camargo, et al.^4^ (2000) and Sculean, et al.^24^ (2003) found greater gain of clinical attachment and bone filling, by means of conventional radiographic analysis, in infra-osseous defects treated with BDX and bovine collagen membrane, when compared to defects treated only with flap surgery. Other studies also used conventional^15^ or digitized^30^ radiographic analysis.

There are few studies involving the treatment of infra-osseous defects by means of RTG with an absorbable barrier, associated or not with other regenerative procedures, which included radiographic subtraction analyses^5,6,8,12,18^. Although the digitized image offers more resources than conventional analysis, such as the possibility of manipulating the image to adjust the gray levels and calculating the quantification of the area of density, as is the case with the conventional method, it consists of subjective analysis. Therefore, it depends on the observer’s visual acuity and degree of prior knowledge, in addition to being subject to interferences of aspects related to the lighting of the room and screen size. Moreover, it does not allow for a precise detection of the change in density between two images^17^.

The radiographic pairs in this study were standardized by means of making individual acrylic film-holders with bite registers for positioning the radiographic film, in order to minimize angular alterations that could cause geometric distortions. It should be pointed out that this standardization consisted of a complex procedure and some geometrical adjustments were made by the computer program used for analysis. These adjustments did not compromise the comparison of images, in such a way that all pairs could be analyzed. Additional care was taken, such as developing by an automatic processor (Perio Pro II), according to the manufacturer’s instructions and under the same conditions. In the majority of cases, this set of procedures allowed for obtaining identical images.

The mean density gains detected by means of subtraction analysis were 0.034 ± 0.423, in the test sites and 0.105 ± 0.423 in the control sites (Table 1). These results were lower than those of other studies in which subtraction analysis was used^5,6,12,18^ to evaluate the results of regenerative therapy. Nevertheless, the time factor must be considered, since the alterations in the degree of bone tissue mineralization are directly related to the alterations in the calcium and phosphate levels, which may require a considerable time to become evident due to biomaterial incorporation and bone remodeling^19^. In the present study, the evaluation period was a time interval of 6 months, while in other studies, the periods of analysis exceeded 6 months^5-8,13,14,18^. This may at least partly explain the lower gain in density observed in this study. Moreover, other differences referring to the position and size of the area of interest in the radiograph may also explain the discrepancy in the results observed in comparison with other studies.

Subtraction analysis demonstrated that the differences between tests and controls were not statistically significant as regards change in density, and as regards quantification in millimeters of the area of alteration (Table 1).

In the present study, no correlation was observed between the alteration in density and the variation in probing depth and attachment level (Table 3). This means that the sites that presented greater increase in density did not correspond to the sites with higher reduction of mean probing depths and clinical attachment gain.

In the study of Christgau, et al.^7^(2006), deep infraosseous defects were treated with b-TCP granules and a bioresorbable membrane. In test defects, autologous platelet concentrate was additionally applied. In both test and controls sites, the clinical attachment gain was accompanied by significant gain in bone density. The radiographic alterations were higher than those of previous studies^3,5,6,8^. Nevertheless, these results must be interpreted with caution because b-TCP is radiopaque and by radiographic methods, one does not distinguish whether it was replaced by vital bone. Other authors have reported that the clinical attachment gain was accompanied by increase in bone density^5,6^. eickholz and Hausmann^12^ (1998) did not find any correlation between attachment gain and bone filling when using digital radiographic analysis. Similarly, eickholz and Hausmann^13^ (2002) found no correlation between gain in bone density measured by subtraction and bone gain measured clinically by the distance between the cementoenamel junction and the base of the bone defect. On the other hand, a study with surgical re-entry^18^ demonstrated that the bone gain, confirmed by measurements made during re-entry, was accompanied by significant gain in bone density detected by radiographic subtraction.

The absence of correlation between bone filling and gain of clinical attachment in the present study should be evaluated with caution. It is important to emphasize that the anatomical characteristics of the bone defect with regard to width and depth may interfere in the gain in bone density observed by subtraction radiography. While the mean bone density evaluates alterations in the buccolingual direction, the gain in attachment consists of a clinical measurement in the coronal apical direction. According to eickholz and Hausmann^13^ (2002), these different measurements may lead to discrepant results, in accordance with the type of defect. In narrow defects, the bone filling promoted by regenerative therapy may result in consistent gain of clinical attachment, with a small gain in bone density. Whereas, in wide defects, greater bone filling of the lateral walls results in consistent gain in bone density, while little or no gain in clinical attachment is observed.

Thus, the absence of correlation between the sites with clinical attachment and bone density gain in this study must not necessarily be interpreted as absence of regeneration. Although some clinical studies have attributed the gain in clinical attachment to the formation of long junctional epithelium^5,8,14,25^, only histological evaluation would be able to determine the type of cure in each regenerative therapy used in this study. If on one hand, the absence of correlation between gain in attachment and bone filling suggests the lack of complete periodontal regeneration, on the other hand, it must be considered the possible influence of the above-mentioned aspects, especially the type of defect, in the analysis of correlation. Nevertheless, as no correlation was established in this study between the characteristics of the defect observed intra-surgically and the gain in density and or the gain in clinical attachment, we are unable to determine the influence of these characteristics on the clinical results. Furthermore, longer periods of evaluation would allow for better evaluation of the effects of regenerative therapy.

## CONCLUSIONS

Within the limitations of the present study, the following conclusions can be pointed out: 1. By the radiographic subtraction analysis, the use of the pool of bovine BMPs in addition to the demineralized lyophilized bovine bone matrix for the treatment of infra-osseous defects did not provide statistically greater gain in bone density; 2. The absence of correlation between improvement in the clinical parameters and the increase in bone density in both groups suggests the lack of occurrence of a complete periodontal regeneration per se. However, longer periods of analysis are required to verify the long-term effect of the therapy; 3. Further studies correlating the characteristics of the bone defect, gain in bone density and gain in probing attachment level would contribute to the evaluation of regenerative therapy in intra-osseous defects.
